# Supplementary material for: Frequency and characteristics of asthma in smokers attending smoking cessation units in Spain
Source: PLoS One. 2023 Dec 7;18(12):e0294587. doi: 10.1371/journal.pone.0294587 (PMC10703326; doi:10.1371/journal.pone.0294587)
Supplement: Attachment — Submitted filename: PNE-D-23-14534_R1-Response to reviewers.docx [file pone.0294587.s002.docx]

PONE-D-23-14534

**Frequency and Differential Characteristics of Asthma in Smokers**

Dear Editor and reviewers,

We would like to thank you for all the constructive comments and suggestions. We really appreciated them and are confident that will clearly contribute to improving the manuscript.

See below our responses (in blue) to your comments (in black) and applied changes.

Looking forward to your response,

Juan Antonio Riesco on behalf of the authors

*********************************************************************************

**REVIEWER #1:**

*General comment*

This is an original paper which assess the frequency and smoking characteristics of asthmatic patients attending 5 different smoking cessation units in Spain.

An adequate number of study subjects is analysed. The topic is of interest for respiratory physicians. The research needs to be improved, particularly in the analysis of lung function and outcome of smoking cessation.

*Specific comments. Major*

- The title should contain information on the fact that the study subjects are smokers attending smoking cessation units.

*We agree with the reviewer; we have modified the title according to his suggestion. The current title is:*

Title page: Frequency and characteristics of Asthma in smokers attending smoking cessation units in Spain.

------------------------------------------------------------------------------------------------------------------------------

- Considering that the study subjects are smokers attending smoking cessation units, the study should include a multivariate analysis assessing the variables possibly associated with smoking cessation at least at short-medium term (e.g. 4-12 weeks from the start of the smoking cessation program) for asthmatic/non-asthmatic smokers.

*Thanks for the comment. The analysis of the factors associated with smoking cessation and possible differences in asthmatics vs non-asthmatics can be very interesting. However, these factors are not the focus of the study and, therefore, information on possible determinants or confounding factors of smoking cessation (such as duration of treatments, adherence, etc.) may be incomplete.*

------------------------------------------------------------------------------------------------------------------------------

- It is unclear if the presented data about the "Smoking cessation interventions" and "Relapse" (Table 4) are related to previous quit attempts or to the actual attempt at the moment of the enrollment in the study.

*We agree that Table 4, on differential characteristics of smoking between asthmatics and non-asthmatics, is difficult to understand. To make it easier to read, we have modified the table by grouping related variables under the same characteristic (in bold type). Thus, the variables related to any quit attempt are grouped together, as well as those referring to the last attempt, regularity of the habit, reward, dependence, or others.*

------------------------------------------------------------------------------------------------------------------------------

- The frequency of airway obstruction in the study subjects should be reported. Obstruction should be defined as FEV1/FVC < 5th percentile of the predicted value, by applying GLI equations (Stanojevic S et al. Eur Respir J 2022)

*The spirometer software has the GLI equations implemented, so the results are already expressed as adjusted. However, we agree on the importance of adding some additional information. In this sense, although GLI recommends a Z score below -1,645, following the recommendations of national and international guidelines, we have calculated the percentage of patients with chronic airflow limitation defined by an FEV1/FVC ratio <0.70 (Page 11).*

------------------------------------------------------------------------------------------------------------------------------

- Discussion section: "We would like to highlight the significantly lower smoking cessation support in asthmatic smokers found in our study." (page 15, lines 263-264). This affirmation should be further clarified in relation to the presented results.

*Thanks for the comment. This sentence is based in results shown in table 4, with a lower proportion of support to quit smoking in asthmatics versus no-asthmatics (60% vs. 77%). On the other hand, we have added some bibliographic reference in the discussion to support this result (page 16).*

------------------------------------------------------------------------------------------------------------------------------

- Discussion section: "However, these patients had significant pulmonary involvement regardless of smoking and did not achieve good smoking cessation rates." (page 15, lines 266-267). This affirmation should be further clarified in relation to the presented results and to those to be added in a revised version of the manuscript.

*The reviewer is right. The sentence is confusing. The idea is to emphasize that the absence of differences in quit rates between asthmatics and no asthmatics probably points to the need to use more intensive intervention strategies in asthmatic smokers because of the impact of smoking on the asthmatic patient's lung function.*

*We have added a short explanation in this regard, as well as bibliography that supports our results (page 16).*

*Minor*
- The abstract lacks a clear aim of the study.

*The reviewer is right, thank you very much for the comment. We have added a sentence with the main purposes of the study (page 2).*

------------------------------------------------------------------------------------------------------------------------------

- "packs per year" should be replaced by: pack-years, throughout the manuscript. In the Material and methods section, it should be reported how "packs per year"/pack-years were calcutated.

*Thank you very much for the comment. We have added a sentence on how to calculate the number of pack-years in the methods section (page 8; line 154). In addition, we have changed the expression “packs per year” by “pack-years” throughout the text, as suggested by the reviewer.*

------------------------------------------------------------------------------------------------------------------------------

- Material and methods section. The used lung function protocol should be reported.

*The reviewer is right. Thank you for your comment. We have added the requested information and referenced it in the relevant section (page 7).*

------------------------------------------------------------------------------------------------------------------------------

- Material and methods section. Reference and description of the UISPM=Test de la Unidad del Instituto de Salud Pública de Madrid should be reported.

*Indeed, we agree that the information on this test is scarce in the article. Consequently, we have added a short explanation of this tool as well as the corresponding bibliography in Methods section (page 8).*

-----------------------------------------------------------------------------------------------------------------------------

- In the title of Table 1, 2, 3 and 4 it should be reported the total number of the study subjects (n=329). Within each table, a note reporting that there were missing data for some indicated variable should be added.

*Certainly, the n changes in different variables due to missing values (that is why the n of each variable was placed in each row). In order to lighten the table, the partial n's have been suppressed and a sentence about the existence of missing values has been added.*

------------------------------------------------------------------------------------------------------------------------------

- "Table 2" is not quoted in the text of the Results section.

*Explanation of table 2 has been quoted in the text (page 10).*

------------------------------------------------------------------------------------------------------------------------------

- Table 2. It should be clarified that Table 2 refers only to the smokers with diagnosis of asthma.

*Thanks for the comment. An explanatory sentence has been added before describing table 2 (page 10).*

------------------------------------------------------------------------------------------------------------------------------

- Table 3:

- Title correct "Tabla 3". *The spelling error has been corrected* *(page 12)*
- Those reported in this table are clinical and respiratory function characteristics in addition to demographic characteristics. The title should be corrected accordingly. *Title has been modified according to the reviewer’s suggestion (page 11).*
- FEV1, FVC, and PEF lack of units of measurement. *Corresponding units have been added to respective parameters in table 3.*

------------------------------------------------------------------------------------------------------------------------------

Numbers and percentages of the obstructed study subjects should be added.

*The chronic air flow limitation, defined by a FEV1/FVC ratio <0.70, has been calculated and added to lung function parameters in table 3.*

------------------------------------------------------------------------------------------------------------------------------

Information on cannabis use is lacking.

*No specific information on cannabis use was collected.*

------------------------------------------------------------------------------------------------------------------------------

- Table 4. "CO levels (exhaled air)" and "HbCo levels" lack of units of measurement.

*The corresponding units have been added to the table (ppm for CO levels and % for HbCO levels) (page 14).*

------------------------------------------------------------------------------------------------------------------------------
